# Diverse, Cryptic, and Undescribed: Club and Coral Fungi in a Temperate Australian Forest

**DOI:** 10.3390/jof11070502

**Published:** 2025-07-03

**Authors:** Vanessa J. McPherson, Michael R. Gillings, Timothy M. Ghaly

**Affiliations:** 1 School of Natural Sciences, Faculty of Science and Engineering, Macquarie University, Sydney, NSW 2109, Australia; timothy.ghaly@mq.edu.au; 2 ARC Centre of Excellence in Synthetic Biology, Macquarie University, Sydney, NSW 2109, Australia

**Keywords:** ITS, barcode, *Ramariopsis*, *Ramaria*, *Clavulinopsis*

## Abstract

Fungi are the most poorly described kingdom of Eukarya. Fundamental questions about their species diversity, their distributions, and their biotic interactions remain largely unanswered, despite fungi playing important roles in the ecology and biogeochemistry of terrestrial ecosystems. To assess some of these data gaps, we intensively surveyed club and coral fungi in a temperate Australian forest in the Upper Lane Cove Valley, Sydney, Australia, over a period of two years. Specimens identified as *Clavulinopsis*, *Ramaria,* or *Ramariopsis* based on morphology were then assigned to operational taxonomic units (OTUs) using the criterion of 97% identity across the entire rDNA internal transcribed spacer (ITS) region. Based on this criterion and ITS-based phylogenies, we identified 80 OTUs in these genera of club and coral fungi within the survey area. Of these OTUs, only 11.25% could be assigned a species name based on BLASTn matches to full-length ITS sequences, suggesting that almost 90% of OTUs were novel taxa, or are yet to be represented in DNA databases. Specimens that were morphologically similar to well-known Northern Hemisphere species were shown to be distinct upon DNA sequencing. Accumulation curves suggest that our surveys only recovered about half of the species in the target genera, and seven times the effort would be required to sample to exhaustion. In summary, even in a small area of less than 100 km^2^, there is evidence for multiple undescribed, cryptic, and undiscovered species. This highlights the fundamental work that remains to be completed in fungal taxonomy and biology.

## 1. Introduction

Fungi are the least well-known group of multicellular eukaryotes. Estimates of the total number of fungi vary widely, ranging between 2.2 and 10 million species. Of this total, there are only about 120,000 valid species names [1,2,3]. Fungi in Australia are poorly known, with at least 75% of species remaining undescribed. This deficit is further complicated by the high levels of endemism in Australian mycobiota [4], the fact that Australian fungi are often named after similar Northern Hemisphere taxa, and that Australia has a large land mass, much of which is unexplored for fungi [5].

It is perhaps not surprising that fungi are under-described, for multiple reasons. Fungi are often cryptic, being microscopic; largely soil dwelling [1,6]; and readily visible only when they produce sporing bodies above ground. Only some species produce macroscopic reproductive structures, with typical mushroom-forming species being better described than those with other fruiting forms, such as the clubs and corals. Sporing bodies are generally small, ephemeral, and difficult to survey in comparison to animals and plants [7]. Expertise in fungal identification is generally declining due to omission of mycology from many university curricula and an aging population of fungal experts [8]. Finally, fungi inhabit diverse niches, many of which are unexplored [9].

Given these problems, DNA-based identification of fungal diversity and fungal species has been proposed as a potential solution. Species identification using DNA barcoding is a powerful tool, applicable to fresh or herbarium specimens and to environmental DNA [10]. The internal transcribed spacer regions of the nuclear ribosomal RNA genes (ITS regions) have been formally recommended as universal DNA barcoding regions for fungi [11]. ITS-based analysis can be used to rapidly process field specimens and generate biodiversity indices [12]. Millions of fungal sequences can be generated in high-throughput sequencing studies to discover new fungal groups and species [13]. Such studies suggest that there might be over 6 million fungal species [1] and have been used to resolve fungal phylogenies [14]. However, molecular data need to be linked to photographs, specimens, and other metadata to be ecologically informative [15,16,17].

We previously conducted extensive field surveys to characterize the diversity of club and coral fungi in a comparatively small river catchment in northern Sydney, Australia. During this work, specimens identified to genera were used to identify biodiversity hotspots [18]. Here, we use fungal barcoding to identify named species and yet-to-be-named taxa in three genera of club and coral fungi that were most commonly recovered during the survey work. Some 90% of specimens could not be matched with known species lodged in DNA databases, demonstrating the undiscovered diversity and cryptic nature of fungal species in these groups, and the fundamental work that still needs to be accomplished to understand fungal biodiversity.

## 2. Materials and Methods

### 2.1. Field Work and Sampling

Field surveys were conducted in the Upper Lane Cove Valley, Sydney, Australia, using the methods outlined in [19]. More than 200 fungal surveys were conducted across the period July 2020 to June 2022 under DPIE Scientific Licence SL 102456. Details of survey methods, locations, and metadata collection are given in [18]. Specimens were stored in 4 mL of 100% ethanol for DNA extraction.

### 2.2. DNA Extraction, PCR, and Specimen Identification

DNA was extracted from 20 mg of ethanol-preserved tissue using a modified salting-out procedure [18,20]. The ITS fungal barcode was amplified from 20 ng of fungal DNA using PCR with primers ITS1 and ITS4 [18,21]. The PCR products were Sanger dideoxy sequenced in both directions by Macrogen, Korea, using the original amplification primers. Sequence files were then edited and contigs generated using GeneStudio v 2.2.0.0. This resulted in 334 full-length ITS sequences from the target genera *Clavulinopsis*, *Ramariopsis,* and *Ramaria.*

### 2.3. Reference Sequences

To generate a reference database of complete ITS sequences, valid fungal species names for each of the target genera were recovered from Species Fungorum (https://www.speciesfungorum.org/, accessed 1 February 2025). Each valid name in these genera was then used to search the NCBI nucleotide database (https://www.ncbi.nlm.nih.gov/nucleotide/, accessed 1 February 2025), using the format ‘*Genus species* ITS’ as the search term. Search results were downloaded for accessions that contained the complete ITS barcode region, which were the partial small subunit rRNA gene, ITS1, 5.8S rRNA gene, ITS2 and partial large subunit rRNA gene. All sequences for Type specimens from the target genera were downloaded from the Fungal Internal Transcribed Spacer RNA (ITS) RefSeq Targeted Loci Project [22]. The final reference database contained 380 full-length ITS sequences. All sequences were confirmed to span the complete ITS1, 5.8S rRNA gene, and ITS2 using ITSx v1.1.3 [23].

### 2.4. Establishing Criteria for Defining Operational Taxonomic Units (OTUs)

**Figure 1:**
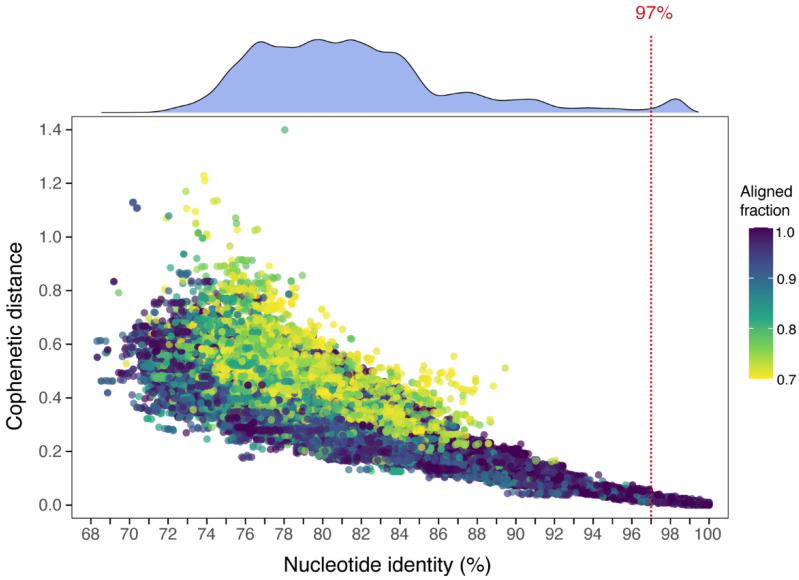
Correlation between Fungal ITS nucleotide identity and phylogenetic distance, plotting pairwise identity between ITS sequences and their phylogenetic (cophenetic) distances. Only comparisons with an ITS alignment coverage >70% are displayed, resulting in the inclusion of >85,000 pairwise comparisons. Points are colored by the aligned fraction of the pairwise sequence alignments. A density histogram of nucleotide identity values is displayed above the plot. The clear nucleotide identity gap between 92% and 97%, suggests a suitable identity threshold of 97% (dashed red line) for fungal OTU clustering of full-length ITS sequences.

Pairwise comparisons were made between all sequences in the combined reference and experimental datasets. Pairwise nucleotide identity comparisons were performed using all-vs.-all sequence alignments with BLASTn v2.15.0 [24]. These pairwise comparisons were reduced to >85,000 comparisons that displayed an alignment coverage of >70%. ITS nucleotide identities were then plotted against their corresponding phylogenetic (cophenetic) distances (Figure 1). For cophenetic distance calculations, a single phylogeny was inferred from all ITS sequences. This involved generating a multiple sequence alignment (MSA) using MUSCLE5 v5.1 [25]. The resulting MSA was trimmed with trimAl v1.4 [26] using the gappyout model. A maximum-likelihood (ML) phylogeny was inferred with IQ-TREE2 v2.2.6 using the best-fit substitution model GTR+F+I+R7, as determined by ModelFinder v1.4.2 [27]. Pairwise cophenetic distances were calculated from the ML tree using the cophenetic.phylo function from the ape R package v4.5.1 [28].

### 2.5. Assigning Sequences into OTUs

A total of 714 full-length ITS sequences (334 Lane Cove and 380 reference GenBank sequences) were clustered into groups. The Leiden clustering algorithm (graph-based) [29] was used to generate clusters with a minimum 97% nucleotide sequence identity and 0.8 aligned fraction across the full-length ITS sequences. Leiden clustering was performed using clusty v1.1.1 [30] with a Leiden resolution parameter of 1.0, a beta value of 0.01, and 10 clustering iterations.

Many of the OTUs defined from field specimens using this method were represented by a single collection (Tables S1–S3). This suggested that the field collections did not represent the complete diversity of OTUs in the study area. To estimate the potential number of undiscovered OTUs, we performed an accumulation analysis. OTU accumulation curves using 1000 random permutations were generated using the iNEXT R package [31], using Chao2 richness estimates as asymptotes, calculated with the specpool function from the vegan R package [32].

### 2.6. Genus Phylogenies

Genus-specific phylogenies were inferred following the methods described above. Briefly, MSAs were generated using MUSCLE5 and trimmed with trimAl’s gappyout model. Phylogenetic trees for each genus were inferred using IQ-TREE2 with the SH-aLRT test and 1000 ultrafast bootstrap replicates, running ModelFinder to determine the best-fit nucleotide substitution model. Trees were visualized using ggtree [33] and ggtreeExtra [34] R packages (Figures S1–S3).

## 3. Results

### 3.1. Nucleotide Diversity and Definition of Operational Taxonomic Units

A total of 741 ITS DNA sequences were obtained from field specimens and from the NCBI nucleotide database. Pairwise nucleotide identities for all ITS sequences, plotted against their corresponding phylogenetic (cophenetic) distances [32], showed that nucleotide identity across the ITS was strongly correlated with evolutionary relatedness (*r^2^* = 0.75, *p* < 2.2 × 10^−16^). Examination of the density histogram (Figure 1) showed a clear nucleotide identity gap between 92% and 97%, indicating a threshold for species delineation. A value of 97% pairwise identity and 0.8 aligned fraction was consequently used to define operational taxonomic units (OTUs), and as a reliable threshold for species-level clustering of full-length ITS sequences. For the purposes of this paper, OTUs were used as a proxy for species.

This cut-off value is supported by the literature on fungal DNA barcoding. In general, fungal specimens with pairwise sequence similarity greater than 97% across the ITS region are taken to be the same species [10,12]. This same identity value has been used to delineate fungal species in metagenomic analyses of environmental DNA samples [1,13]. For some groups, such as yeasts, the cut-off is higher, at 98.4% [35].

A total of 248 OTUs were defined in the entire dataset, based on clustering of ITS sequences into groups exhibiting greater than 97% pairwise similarity. The Lane Cove field specimens generated 80 OTUs, of which 71 were comprised only Lane Cove sequences. Consequently, only nine OTUs (11.25%) from field specimens could be assigned species names based on 97% nucleotide identity to full-length ITS reference sequences (Table 1). This could be a result of the unmatched OTUs not having a full-length ITS sequence represented in the database. To address this possibility, ITS sequences from each of the unidentified OTUs were used to interrogate the NCBI nucleotide database using BLASTn (megablast) (https://blast.ncbi.nlm.nih.gov/, accessed 1 February 2025), excluding uncultured/environmental sample sequences. Search results were scrutinized to identify sequenced specimens to species where possible, resulting in some additional assignments of potential species names (Tables S1–S3).

**Figure 2:**
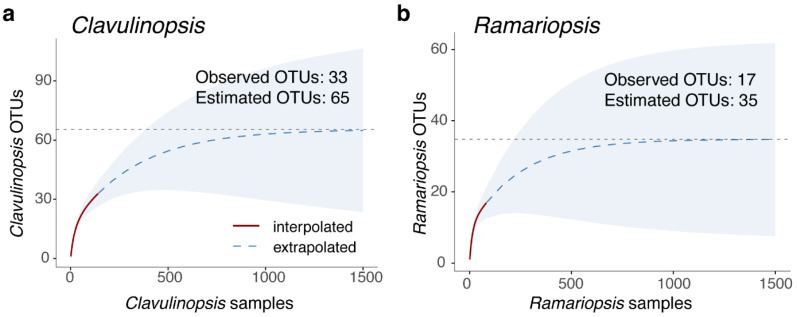
Fungal OTU accumulation curves with extrapolation. Accumulation curves are shown for (**a**) *Clavulinopsis* and (**b**) *Ramariopsis*. For each plot, the solid red line represents interpolated OTU accumulation (observed sampling effort), while the dashed blue line shows extrapolation to 1500 fungal samples. The shaded region indicates 95% confidence intervals derived from 1000 bootstrap replications. The dotted horizontal line marks the asymptotic richness estimate (Chao2).

Many of the OTUs generated from field specimens were represented by a single collection (Tables S1–S3). This suggested that the field collections did not represent the complete diversity of OTUs in the study area. To estimate the potential number of undiscovered OTUs, we performed an accumulation analysis (Figure 2). Such analysis showed that only about 50% of the OTUs might have been recovered, even though over 200 separate surveys were conducted (Figure 2). Accumulation curves for *Ramaria* are not presented because they had unacceptable error bars, likely caused by the large number of singleton OTUs in the *Ramaria* collections. This phenomenon is possibly driven by a high underlying diversity and sporadic fruiting in this genus.

For the three target genera, there were 424 valid species names listed in Index Fungorum. Of these, only 128 (30%) were represented by full-length ITS sequences in the NCBI database. Using the criterion of 97% pairwise nucleotide identity, ITS sequences from 103 of 128 reference sequences from named species fell into a single OTU, largely confirming the utility of the 97% cut-off value (Table 1). Exceptions to this rule fell into two broad categories: NCBI accessions that were clearly mis-annotated based on their appearance in distant sections of the phylogenies (Figures S1–S3), and sets of OTUs that clustered in the phylogenies, likely reflecting species complexes and genetic divergence in species groups with wide distributions (Tables S1–S3).

### 3.2. Clavulinopsis Diversity

A total of 142 *Clavulinopsis* field specimens were successfully sequenced across the entire ribosomal RNA gene internal transcribed spacer (ITS) region. These sequences clustered into 33 distinct groups based on each group sharing more than 97% nucleotide identity across the ITS region (Table 1). Groups sharing more than 97% ITS identity were defined as distinct OTUs, equivalent to species for the purposes of this paper (Figure 1).

Of the 33 *Clavulinopsis* OTUs from field collections, about half were represented by only a single sequenced specimen (Table S1). This suggested that the field collections were far from a complete representation of the *Clavulinopsis* species present, and a significant number of additional OTUs were still to be discovered, even within the comparatively small area of the Lane Cove Valley (95.4 km^2^). An accumulation curve for *Clavulinopsis* had a predicted asymptote at 65 OTUs (Figure 2a), suggesting only half of the *Clavulinopsis* diversity was sampled during our field campaign, despite sampling on over 200 days.

To assign species names to field specimens, ITS sequences were aligned with reference sequences downloaded from NCBI. Only 6 of the 33 field OTUs (18.2%) exhibited greater than 97% nucleotide identity to named reference sequences downloaded from NCBI (Table 1). The species names that could be assigned to field specimens on this basis included *Clavulinopsis amoena*, *antillarum*, *fusiformis*, *laeticolor*, *sulcata,* and *trigonospora* (Table S1). The Australian Fungi List (https://fungi.biodiversity.org.au/nsl/services/search/taxonomy, accessed 1 February 2025) names a total of 26 *Clavulinopsis* species in Australia. Here we recorded 33 OTUs of which 6 could be assigned names, thus increasing the number of potential *Clavulinopsis* species in Australia by 40–100%, despite surveying less than 0.001% of the Australian continent.

Of the named species, *C. antillarum* and *C. trigonospora* are not in the Australian Fungal List (AFL). *C. antillarum* [36] has previously been reported from Tasmania, Australia, as a DNA sequence, NCBI accession HQ877710 [37]. One of our *Clavulinopsis* specimens (LC_1741) matched the Type specimen of *C. trigonospora* from Italy [38] and sequences subsequently reported from China [39]. Consequently, this paper becomes the first record of the occurrence of *C. trigonospora* in Australia.

The four most commonly reported *Clavulinopsis* species in Australia are *C. amoena*, *C. sulcata*, *C. corallinorosacea,* and *C. fusiformis* (https://inaturalist.ala.org.au/, accessed 1 February 2025). Of these, only *C. sulcata* was commonly identified in our collections, and *C. corallinorosacea* was not detected at all using the 97% identity criterion, despite a number of field specimens conforming to the general morphology reported for this species. Indeed, specimens with morphologies typical of *C. corallinorosacea* fell into multiple OTUs with relatively distinct nucleotide sequences.

**Figure 3:**
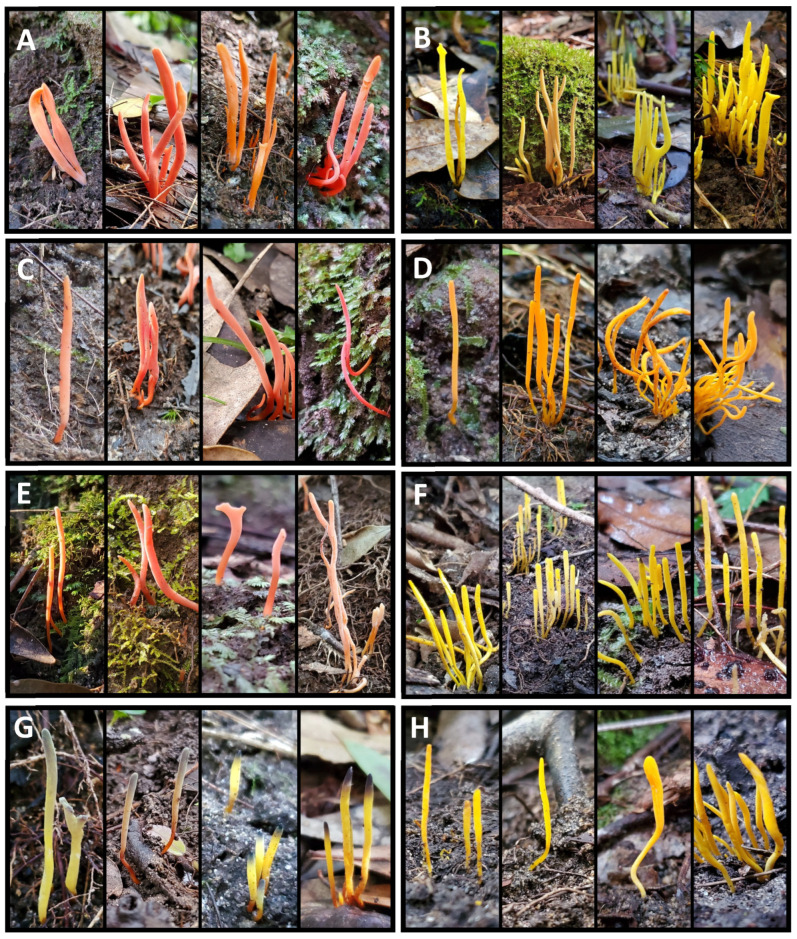
Representative spore bodies of *Clavulinopsis* specimens encompassing the OTUs that were most commonly recovered during this work. (**A**): OTU35 *Clavulinopsis sulcata*; (**B**): OTU20 *C. antillarum*; (**C**): OTU10; (**D**): OTU17 *C. laeticolor*; (**E**): OTU7; (**F**): OTU12; (**G**): OTU3; (**H**): OTU4. Approximately two-thirds life size.

Specimens that represent the morphologies of the *Clavulinopsis* OTUs most commonly recovered in our surveys are presented in Figure 3. Specimens identified as OTU35 *C. sulcata* (Figure 3A) could not be distinguished in the field from specimens assigned to OTU10 and OTU7 (Figure 3C,E), even though these OTUs were placed into distant parts of the *Clavulinopsis* phylogeny (Figure S1). Similarly, the yellow clubs OTU20 *C. antillarum*, OTU12, and OTU4 (Figure 3B,F,H) could not be easily distinguished. Thus, identification based on morphology is likely to significantly underestimate the diversity of *Clavulinopsis* species. Two commonly encountered OTUs could be identified in the field, OTU17 *C. laeticolor*, based on its orange coloration (Figure 3D), and the unnamed OTU3, which has distinctive blue-gray tips on a yellow club (Figure 3G).

Examining reference sequences from NCBI, it is apparent that there are potential mis-annotations in the dataset. For instance, sequences annotated as *C. fusiformis* in the NCBI database fell into seven different OTUs (Table S1), and these OTUs were scattered across the *Clavulinopsis* phylogeny (Figure S1), showing that they were not members of a diverging species complex. A total of seven named *Clavulinopsis* ‘species’ lodged in NCBI were each assigned to multiple OTUs, demonstrating that *C. fusiformis* was not an exceptional case in this regard (Table 1). Again, identification based solely on morphology is likely to lie behind these mis-annotations.

### 3.3. Ramariopsis Diversity

A total of 83 *Ramariopsis* specimens were sequenced across the ITS region. Based on sharing at least 97% nucleotide identity across the ITS region, these specimens clustered into 17 OTUs (Table 1). Again, these OTUs probably do not represent the full *Ramariopsis* diversity within the study area, since the accumulation analysis suggests an additional 18 OTUs are likely to be present (Figure 2b).

ITS sequences were aligned with reference sequences downloaded from NCBI. Only 2 of the 17 OTUs (11.8%) exhibited greater than 97% nucleotide identity to reference sequences (Table 1). The species names assigned to field specimens on this basis included *Ramariopsis bicolor* and *R. avellano-inversa*. These species are not in the AFL (https://fungi.biodiversity.org.au/nsl/services/search/taxonomy, accessed 1 February 2025). *R. bicolor* has been reported from New Zealand [40], and the three specimens here cluster adjacent to New Zealand accessions in the phylogeny (Figure S2). *R. avellano-inversa* has also been recorded from New Zealand and from Italy [38], and one specimen in our collection clustered with the Italian accessions. On this basis, the current paper represents the first records of *R. bicolor* and *R. avellano-inversa* in Australia.

**Figure 4:**
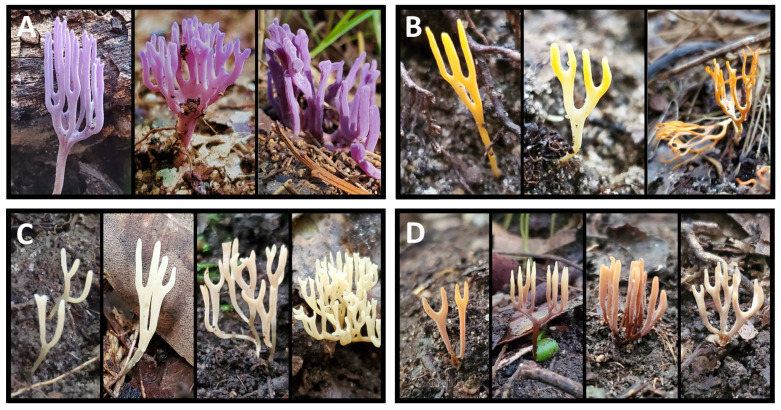
Representative spore bodies of *Ramariopsis* specimens encompassing the OTUs that were most commonly recovered during this work. (**A**): OTU214, a purple coral similar to *R. pulchella*, but distinct upon DNA analysis; (**B**): OTU215, an orange-yellow coral similar to *R. crocea*, but distinct upon DNA analysis; (**C**): OTU216, a white coral; (**D**): OTU221, a tan-brown coral. Approximately two-thirds life size.

Some of our specimens had distinct morphologies that would normally allow them to be confidently identified in the field. For instance, OTU214 included small purple-blue corals, morphologically similar to *R. pulchella* (Figure 4A). However, multiple reference sequences for *R. pulchella* clustered adjacent but separately from OTU214 (Figure S2). Similarly, orange-yellow corals with similar morphology to *R. crocea* (OTU215, Figure 4B) were distinct from reference sequences of this species (Figure S2).

Consequently, two apparently easily identifiable *Ramariopsis* are actually not these species in Australia, exhibiting significant DNA divergence from the well-known taxa. These unnamed OTUs also have considerable morphological diversity in coloration and branching patterns, as do OTUs 216 and 221 (Figure 4).

The two most commonly recovered groups of *Ramariopsis*, OTU216 and OTU221 (Figure 4C,D), nested within clades entirely composed of OTUs recovered during this study. These clades have no closely related reference sequences, and form isolated clusters of species within the *Ramariopsis* phylogeny (Figure S2). This suggests that there are several novel radiations of *Ramariopsis* unique to the Australian mycobiome.

Again, the NCBI reference sequences included potential mis-annotations. Accessions for *R. pulchella*, *R. minitula,* and *R. kunzei* could be found in different parts of the phylogeny (Figure S2) and fell into multiple OTUs (Table S2). Identification of specimens based solely on morphology could lie behind these mis-annotations, but with such distinctive species as *R. pulchella* included, incorrect labeling is perhaps more likely in some cases.

### 3.4. Ramaria Diversity

*Ramaria* is a large and complex genus of robust coral fungi. Full-length *Ramaria* ITS sequences downloaded from NCBI were classified into 102 OTUs based on the 97% matching criterion. These sequences were annotated as 89 distinct species (Table S3). Where named species comprised multiple OTUs, this likely occurred as a consequence of misidentification, mislabeling, or diverging species complexes, as outlined above.

In contrast, eight OTUs contain two named species. In these cases, ITS sequences might not resolve the taxa, with the 97% nucleotide identity cut-off being too broad. Alternatively, the species might be the same taxon, named differently in different parts of their distributions. These questions could be resolved via sequencing of additional barcodes such as the genes for translation elongation factor 1-α (*TEF1α*) or RNA-polymerase II subunit (*RPB2*) [41,42].

There are 33 *Ramaria* species named in the Australian Fungal List, and 11 of these were represented by sequences in the reference collection. Upon analysis of ITS sequences from our field specimens, 30 OTUs were defined. Only one of the field specimens could be assigned a species name by matching with the reference sequence collection. This was OTU98 *R. anziana* (Table S3), a species that has previously been reported from Australia [43].

Less than 3.33% of *Ramaria* field specimens could be assigned a species name based on nucleotide matches, despite the large number of sequences in our reference set and specimens in the collection. It could be that some species were missing from the reference set because they were not represented by full-length ITS sequences. To address this concern, we performed BLASTn searches for highly similar sequences (megablast) on all the unidentified OTUs through the NCBI BLAST portal. We excluded environmental sequences in this search. This approach did identify additional field OTUs, but did not significantly change the general results. OTU92 matched accession KY352642.1, a *Ramaria sp*. from Argentina, and OTU99 showed 99% similarity to accession JX310378.1, *R. abietina* from the USA (Table S3).

The four most commonly reported *Ramaria* species on iNaturalist Australia (https://inaturalist.ala.org.au/observations, accessed 1 February 2025) are *R. filicicola, R. lorithamnus, R. capitata,* and *R. anziana*, collectively accounting for about 25% of records. Of these, only *R. anziana* could be identified in our collections using nucleotide matches to reference sequences. It is possible that the three other species are simply not represented in nucleotide databases. There is a full-length ITS sequence available for *R. lorithamnus* (accession HQ533039.1), but none of our OTUs matched this sequence, and consequently, *Ramaria* specimens morphologically similar to *R. lorithamnus* in Australia are not the same species as the New Zealand specimen sequenced in that accession. Neither *R. filicicola* nor *R. capitata* have representative ITS sequences in NCBI, despite being known to occur in Australia [43,44]. Examining the morphology of our two most common OTUs for *Ramaria*, specimens from OTU 87 match the descriptions of *R. capitata* (=*ochraceosalmonicolor*) [43]. Specimens from OTU 96 match *R. filicicola*, exhibiting the distinctive rhizomorphs of this species [44].

## 4. Discussion

Fungi are critically important members of ecosystems [45], and yet they are the least well characterized of all eukaryotes [9,46]. Even the extent of our ignorance about fungi is unknown. Estimates of the number of fungal species range widely, between 1 and 10 million, and recent analyses concluded that there was not enough evidence to arrive at a consensus [3,47,48]. What is needed is a concerted, global effort that unites field work with high-throughput DNA analyses.

To help understand the magnitude of the challenge and identify some of the key impediments to such work, we used a temperate forest in the north of Sydney, Australia, as a test case. Here we present the results of a DNA barcoding study for three of the most commonly recovered genera of club and coral fungi, *Clavulinopsis*, *Ramariopsis,* and *Ramaria.*

**Most fungal specimens from the area are novel taxa.** Here we empirically tested the nucleotide sequence similarity across the full-length ITS region that could delineate OTUs as a proxy for species. A value of 97% similarity was determined (Figure 1), in agreement with other analyses [1,10,12,13]. Based on this criterion, only 11.25% of our OTUs could be matched with sequences from named species in DNA databases. This suggests that some 89% of OTUs could be novel taxa. Previous estimates suggest that 75% of Australian fungi are undescribed [5] and ITS barcoding of South American fungal fruiting bodies generated 70% unmatched OTUs [12]. Edible wild mushrooms from China were unmatched to databases over 50% of the time [49].

Our novel OTUs are on the high side of published values, and this might in part be due the fact that many Australian species are, as yet, unrepresented in DNA databases. DNA sequence data are available for about 45,000 fungal species, and a large proportion of these data are ITS accessions [16]. Nevertheless, given that there are millions of fungal species [48], these data might represent only 1–2% of the species in existence [16]. Nevertheless, it is likely that the majority of our OTUs are novel taxa.

**Much more surveying is needed to recover all taxa.** Many of the OTUs we defined were represented by single specimens, suggesting our surveys were not complete. Accumulation curves predicted that we recovered only half the OTUs in the survey area (Figure 2). Despite conducting over 200 days of surveying, up to seven times the effort might be needed to sample to exhaustion. In addition, our survey area was comparatively limited, at less than 100 km^2^, comprising only 0.001% of the Australian continent. This gives an idea of the scale of effort that would be needed to characterize fungal diversity in Australia.

We know that fungi exhibit biogeographic patterns and that species composition changes with distance [50]. Soil fungi are endemic to particular bioregions [51], and species composition might be climate-driven [52] rather than being primarily driven by plant or animal associations [4,53]. Consequently, surveys of thousands of locations similar in size to that reported here might be needed to obtain a snapshot of fungal diversity in Australia. Although estimates vary, there could be 250,000 species of fungi in Australia, of which 90% might be endemic [54]. Perhaps 75% of Australian species remain undescribed [5]. To begin this task of description, regions of high endemicity should be targeted. These include the NE and NW savannas, SW woodlands, and the SE temperate forests [46].

**There is evidence for unique Australian radiations in the target genera.** Australia has been largely isolated during its geological history, resulting in a megadiverse and endemic biota [55]. The unique nature of Australia’s plants and animals is likely to be repeated in the fungi of Australia. However, Australian fungi have often been named after morphologically similar Northern Hemisphere counterparts [5], thus obscuring the diversity of the Australian mycobiome.

In this study, we found multiple examples of instances where specimens that would ordinarily be assigned to species based solely on morphology were shown to be distinct upon DNA analysis. For instance, two distinctive species, *Ramariopsis pulchella* and *Ramriopsis crocea,* were shown to have related but genetically distinct representatives in Australia (Figure 4). Further, within the *Ramariopsis* phylogeny, there were clusters of uniquely Australian OTUs that formed at least two distinct clades (Figure S2). These clades contain the two most commonly recovered OTUs, 216 and 221. This possibly indicates that there could be two independent radiations of *Ramariopsis* within Australia. Increased sampling effort could help confirm this suggestion. Similar instances of uniquely Australian clades can be found in the *Clavulinopsis* and *Ramaria* phylogenies (Figures S1 and S3).

**Field identification is difficult because of cryptic species and within-species phenotypic variation.** DNA barcoding and definition of OTUs allows genetically different taxa to be unequivocally distinguished. However, many distinct OTUs recovered in our surveys had similar or overlapping phenotypes, even though they occupied distant places in the phylogenies. For example, red clubs assigned to *Clavulinopsis* were not possible to distinguish based solely on appearance (Figure 3). The same can be said of yellow clubs in the same genus.

Morphologically indistinguishable but nevertheless distinct taxa are known as cryptic species. It has been estimated that such cryptic species might comprise a large proportion of undiscovered fungal diversity [56]. There are good examples of cryptic species complexes in Australian taxa [57], and cryptic species cause complications in understanding fungal ecology, diversity, and taxonomy [58].

In contrast, specimens belonging to the same OTU often exhibited variable morphology in color, form, and fruiting body arrangement. Figure 3 and Figure 4 illustrate significant morphological differences within specimens assigned to single OTUs. This phenotypic plasticity often exceeds the morphological differences typically used to separate species, and further complicates species identifications without sequencing analysis [58].

**Validly named fungal species often have no sequence representatives in databases.** While assembling our reference collection of ITS sequences, we used Species Fungorum as a source of validly named species. Of the 424 valid species listed for *Clavulinopsis, Ramariopsis,* and *Ramaria*, only 128 (30%) were represented by full-length ITS sequences in NCBI. This proportion is typical of fungi more generally. It is widely reported that only 30% of known species have DNA sequences available [16,48]. This limits the efficacy of BLAST searches to help identify unknown specimens. Linking OTUs to species names will be an ongoing task into the future. The clear solution to this problem is an increased sequencing effort, particularly focused on yet-to-be-sequenced vouchers and Type specimens [10,17,59].

**Fungal DNA sequences in databases are often mis-annotated.** A significant proportion of the reference sequences for particular species that we downloaded from NCBI fell into multiple OTUs (20%, Table 1). On closer examination, many of these sequences were shown to lie in distant parts of their respective phylogenies (Figures S1–S3), and consequently were not all members of the same species. Such mis-annotations are common, and in general, about 20% of nucleotide accessions are incorrectly identified at species level across the fungi [60], in agreement with our findings.

Targeted and intensive DNA sequencing of voucher specimens, coupled with quality control and clustering analysis, will help resolve this problem and expand the coverage of sequence data [61]. Construction of detailed phylogenies of the fungi will also help define taxonomically coherent groups [14]. A number of curated databases have been established that will greatly enhance the identification of fungi using sequence data [59]. These include UNITE [62,63], the ISHAM DNA Barcoding database [64], and RefSeq (targeted loci) [22].

## 5. Conclusions

Fungi are key components of terrestrial biomes, participating in diverse symbioses and cycling nutrients through ecosystems. They have a central role in maintaining biodiversity and plant productivity. However, knowledge of fungi and their interactions is sparse in comparison to animals and plants. Part of this problem is driven by the cryptic nature of fungi but also by the paucity of mycologists in comparison to zoologists and botanists [8]. One of the best things we could do is to include more mycology in undergraduate and postgraduate curricula, with the aim of increasing the number of researchers with an interest in fungi.

We are only just beginning to outline the extent of fungal diversity, that is, assembling lists of the fungal species that drive ecosystem processes. Managing these processes will rely on an understanding of the fungi in any ecosystem. We know that fungi exhibit endemism [4,51], and the forces behind their distributions and diversity are being discovered [46]. Given that climate is a major driver of fungal distribution [52], understanding fungal composition and fungal activity in ecosystems will be important for a sustainable future under a changing climate [65].

## Figures and Tables

**Table 1:** Summary of ITS sequencing data.

Genus	Source ^A^	#ITS Sequences ^B^	#OTUs ^C^		#Specimen OTUs Matched ^D^	#Ref Species ^E^	Refs with Multiple OTUs ^F^
*Clavulinopsis*	Field	142	33		6 (18.2%)		
	NCBI	110		46		25	7 (28.0%)
*Ramariopsis*	Field	83	17		2 (11.8%)		
	NCBI	33		20		14	3 (21.4%)
*Ramaria*	Field	109	30		1 (3.33%)		
	NCBI	237		102		89	16 (18.0%)
**Total**		714	80	168	9 (11.25%)	128	25 (20.3%)

A: ITS DNA sequences were generated from field specimens or were downloaded from NCBI; B: Number of full-length ITS sequences analyzed; C: Number of operational taxonomic units (OTUs) defined by >97% nucleotide similarity across the ITS region; D: Number of field specimen OTUs able to be matched with reference sequences at >97% nucleotide identity. Most field specimens were unable to be identified using ITS sequences from NCBI; E: Number of named species in the reference dataset; F: Number of named species in the reference dataset that were assigned to multiple OTUs.

## Data Availability

DNA sequences reported in this paper are lodged as GenBank accession numbers PV827770–PV828102. Voucher specimens of representative OTUs have been lodged in the Downing Herbarium at Macquarie University with accession numbers in the range MQU71000150–MQU71000700.

## Supplementary Materials

The following supporting information can be downloaded at https://www.mdpi.com/article/10.3390/jof11070502/s1, Figure S1: *Clavulinopsis* ITS phylogeny; Figure S2: *Ramariopsis* ITS phylogeny; Figure S3: *Ramaria* ITS phylogeny; Table S1: Operational taxonomic units for specimens and reference sequences in the genus *Clavulinopsis*; Table S2: Operational taxonomic units for specimens and reference sequences in the genus *Ramariopsis*; Table S3: Operational taxonomic units for specimens and reference sequences in the genus *Ramaria.*
