# Supplementary material for: Diverse, Cryptic, and Undescribed: Club and Coral Fungi in a Temperate Australian Forest
Source: J Fungi (Basel). 2025 Jul 3;11(7):502. doi: 10.3390/jof11070502 (PMC12298858; doi:10.3390/jof11070502)
Supplement: Supplementary file 1 [file jof-11-00502-s001.zip › Figure S1 Clavulinopsis tree.pdf]

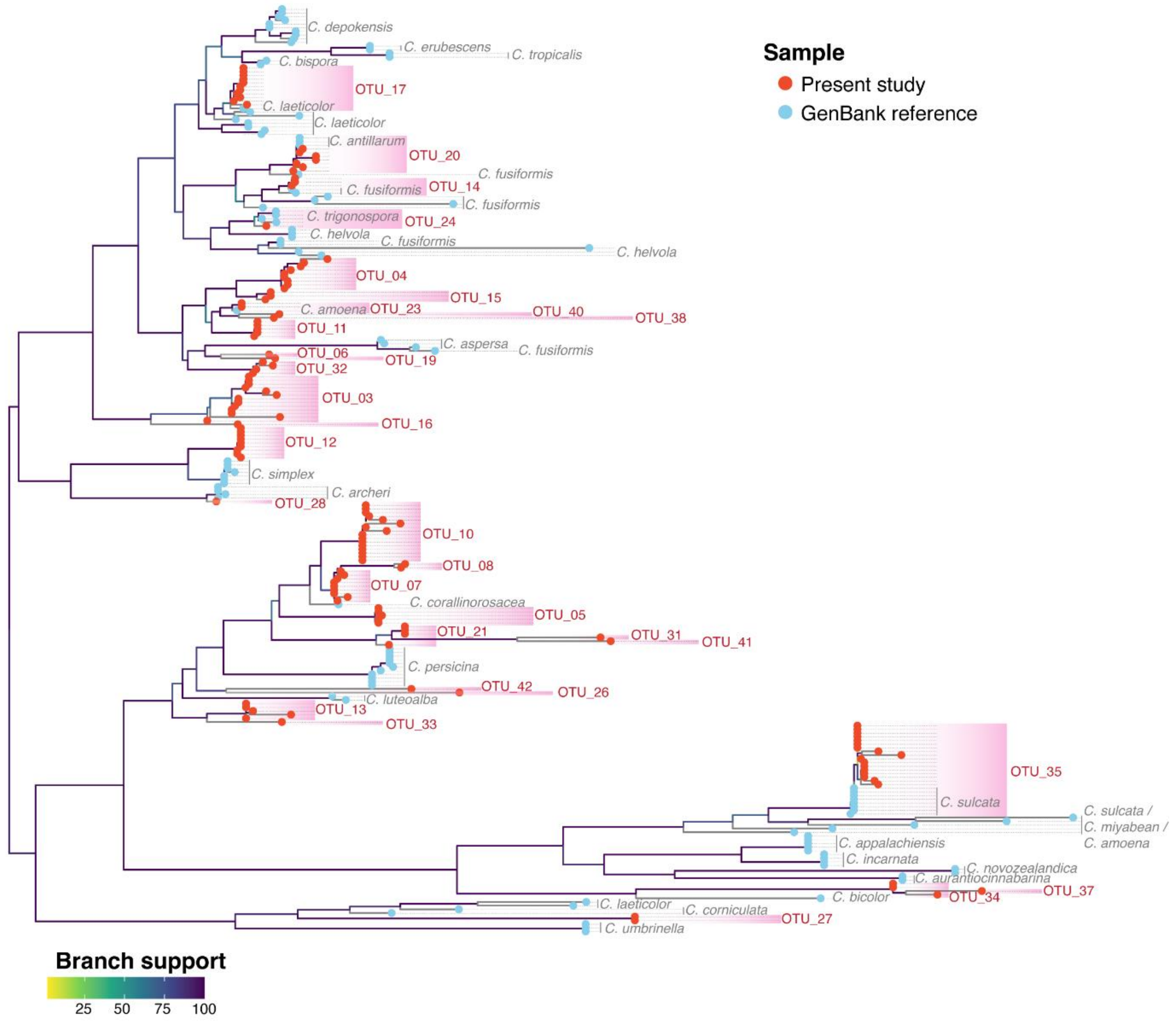

Figure S1. *Clavulinopsis* ITS phylogeny. A maximum-likelihood phylogeny (GTR+F+R4 nucleotide substitution model) based on full-length ITS sequences derived from the *Clavulinopsis* genus. The tree was rooted using two Ustilaginomycotina species as outgroup taxa, *Tilletia puccinelliae* (NCBI accession MH863261.1) and *Malassezia restricta* (AY743636.1), which were subsequently removed after rooting. Branch colours (colour scale bar) indicate the degree of branch support based on 1,000 ultrafast bootstrap (UFBoot) replicates. Pink boxes indicate fungal OTUs recovered in the present study (red tippoints), based on 97% fungal ITS nucleotide identity. Labelled taxa (blue coloured tippoints) indicate GenBank reference ITS sequences.
