# Supplementary material for: Diverse, Cryptic, and Undescribed: Club and Coral Fungi in a Temperate Australian Forest
Source: J Fungi (Basel). 2025 Jul 3;11(7):502. doi: 10.3390/jof11070502 (PMC12298858; doi:10.3390/jof11070502)
Supplement: Supplementary file 1 [file jof-11-00502-s001.zip › Table S1 Clavulinopsis OTUs.pdf]

Table S1: Operational taxonomic units for specimens and reference sequences in the genus *Clavulinopsis*. OTUs were defined based on sharing a minimum of 97% nucleotide sequence identity across the complete rRNA internal transcribed spacer region (see Fig 1).

Operational taxonomic unit[A]	# Field Specimens	# matching NCBI Ref Sequences	Matching NCBI species[B]	Duplicate NCBI annotations[C]
3	12	0		
4	9	0		
5	5	0		
6	1	0		
7	9	0	(96.2% *C. corallinorosacea* KP25744.1)	
8	2	0		
10	16	0		
11	5	0	(95.8% *C. corallinorosacea* KP25744.1)	
12	9	0	(96.5% *C. simplex* OR567669.1 NZ)	
13	5	0		
14	3	2	***C. fusiformis***	OTUs 22,46,53,54,55,56
15	3	0		
16	1	0		
17	11	1	***C. laeticolor***	OTUs 75,76,77,78,79,80,81
18	1	0		
19	1	0		
20	7	3	***C. antillarum***	
21	4	0	(97.5% *C. sp.* OR567705.1 NZ)	
23	2	1	***C. amoena***	OTU 43
24	1	4	***C. trigonospora***	
26	1	0		
27	2	0		
28	1	0		
31	1	0		
32	4	0		
33	1	0	(99.65% *C. sp.* OR567600.1 NZ)	
34	3	0	(96.11% *C. sp.* PQ346228.1 USA)	
35	17	8	***C. sulcata***	OTUs 67,68
37	1	0		
38	1	0	(95.29% *C. amoena* OR567698.1 NZ)	
40	1	0		
41	1	0		
42	1	0		
43	0	1	***C. amoena***	OTU 23,
44	0	5	***C. appalachiensis***	
29	0	4	***C. archeri***	
45	0	3	***C. aspersa***	
46	0	1	***C. fusiformis***	OTUs 14,22,53,54,55,56
47	0	2	***C. aurantiocinnabarina***	
22	0	1	***C. fusiformis***	OTU 14,46,53,54,55,56
48	0	1	***C. bicolor***	
49	0	2	***C. bispora***	
50	0	1	***C. corallinorosacea***	
51	0	10	***C. depokensis***	
52	0	2	***C. erubescens***	
53	0	1	***C. fusiformis***	OTUs 14,22,46,54,55,56
54	0	2	***C. fusiformis***	OTUs 14,22,46,53,55,56
55	0	2	***C. fusiformis***	OTUs 14,22,46,53,54,56
56	0	1	***C. fusiformis***	OTUs 14,22,46,53,54,55
57	0	1	***C. helvola***	OTUs 58,59,74
58	0	1	***C. helvola***	OTUs 57,59,74
59	0	3	***C. helvola***	OTUs 57,58,74

60	0	4	***C. incarnata***	
61	0	2	***C. luteoalba***	
62	0	1	***C. miyabeana***	OTU 63
63	0	1	***C. miyabeana***	OTU 62
64	0	2	***C. novozealandica***	
65	0	11	***C. persicina***	
66	0	7	***C. simplex***	
67	0	1	***C. sulcata***	OTUs 35,68
68	0	1	***C. sulcata***	OTUs 35,67
69	0	2	***C. tropicalis***	
70	0	3	***C. umbrinella***	
72	0	1	***C. corniculata***	OTU 73
73	0	1	***C. corniculata***	OTU 72
74	0	1	***C. helvola***	OTUs 57,58,59
75	0	2	***C. laeticolor***	OTUs 17,76,77,78,79,80,81
76	0	1	***C. laeticolor***	OTUs 17,75,77,78,79,80,81
77	0	1	***C. laeticolor***	OTUs 17,75,76,78,79,80,81
78	0	1	***C. laeticolor***	OTUs 17,75,76,77,79,80,81
79	0	2	***C. laeticolor***	OTUs 17,75,76,77,78,80,81
80	0	1	***C. laeticolor***	OTUs 17,75,76,77,78,79,81
81	0	1	***C. laeticolor***	OTUs 17,75,76,77,78,79,80

A: OTUs defined on the basis of 97% nucleotide identity across the complete ITS region; B: Names in brackets indicate results of Blastn matches to partial ITS sequences in NCBI. Environmental sequences excluded; C: Instances where species were assigned to multiple OTUs are listed.
