# Supplementary material for: Diverse, Cryptic, and Undescribed: Club and Coral Fungi in a Temperate Australian Forest
Source: J Fungi (Basel). 2025 Jul 3;11(7):502. doi: 10.3390/jof11070502 (PMC12298858; doi:10.3390/jof11070502)
Supplement: Supplementary file 1 [file jof-11-00502-s001.zip › Table S2 Ramariopsis OTUs.pdf]

Table S2: Operational taxonomic units for specimens and reference sequences in the genus *Ramariopsis*. OTUs were defined based on sharing a minimum of 97% nucleotide sequence identity across the complete rRNA internal transcribed spacer region (see Fig 1).

Operational taxonomic unit[A]	# Field Specimens	# matching NCBI Ref Sequences	Matching NCBI species[B]	Duplicate NCBI annotations[C]
9	1	0		
25	2	0		
30	1	0		
214	5	0	(97% *R. 'pulchella'* KP012919 AUST)	
215	5	0	(98% *R. sp.* OR567564 NZ)	
216	19	0		
217	4	0		
218	8	0	(98.2% *R. sp.* MH930389.1 CHILE)	
219	6	0		
220	6	0		
221	14	0		
222	1	0		
223	5	0		
224	3	2	***R. bicolor***	
225	1	0		
226	1	1	***R. avellaneo-inversa***	
227	1	0		
229	0	1	***R. atlantica***	
230	0	2	***R. avellanea***	
231	0	3	***R. crocea***	
232	0	1	***R. flavescens***	
233	0	3	***R. gilbertoi***	
234	0	2	***R. hirtipes***	
235	0	1	***R. kunzei***	OTUs 236,237
236	0	2	***R. kunzei***	OTUs 235,237
237	0	1	***R. kunzei***	OTUs 235,236
238	0	1	***R. minutula***	OTU 239
239	0	1	***R. minutula***	OTU 238
240	0	1	***R. pulchella***	OTUs 241,242,243
241	0	1	***R. pulchella***	OTUs 240,242,243
242	0	1	***R. pulchella***	OTUs 240,241,243
243	0	1	***R. pulchella***	OTUs 240,241,242
244	0	5	***R. ramarioides***	
245	0	1	***R. robusta***	
246	0	2	***R. subtilis***	

A: OTUs defined on the basis of 97% nucleotide identity across the complete ITS region; B: Names in brackets indicate results of Blastn matches to partial ITS sequences in NCBI. Environmental sequences excluded; C: Instances where species were assigned to multiple OTUs are listed.
