# Supplementary material for: Diverse, Cryptic, and Undescribed: Club and Coral Fungi in a Temperate Australian Forest
Source: J Fungi (Basel). 2025 Jul 3;11(7):502. doi: 10.3390/jof11070502 (PMC12298858; doi:10.3390/jof11070502)
Supplement: Supplementary file 1 [file jof-11-00502-s001.zip › Table S3 Ramaria OTUs.pdf]

Table S3: Operational taxonomic units for specimens and reference sequences in the genus *Ramaria*. OTUs were defined based on sharing a minimum of 97% nucleotide sequence identity across the complete rRNA internal transcribed spacer region (see Fig 1).

Operational taxonomic unit[A]	# Field Specimens	# matching NCBI Ref Sequences	Matching NCBI species[B]	Duplicate NCBI annotations[C]
82	9	0		
83	7	0		
84	8	0		
85	5	0		
86	1	0		
87	23	0		
88	2	0		
89	2	0		
90	7	0		
91	2	0		
92	1	0	(97.14% *R. sp.* KY352642.1 Argentina)	
93	1	0		
94	1	0		
95	1	0		
96	12	0		
97	1	0		
98	5	1	***R. anziani***	
99	2	0	(98% *R. abietina* JX310378.1 USA)	
100	1	0		
101	2	0		
102	2	0		
104	1	0		
105	1	0		
106	1	0		
107	2	0		
108	3	0		
109	1	0		
110	2	0		
111	2	0		
112	1	0		
113	0	4	***R. acrisiccescens***	
114	0	1	***R. admirata***	
115	0	1	***R. albidoflava***	
116	0	1	***R. amyloidea***	OTU 117
117	0	1	***R. amyloidea***	OTU 116
118	0	9	***R. apiculata***	Also *R. tsugina*
119	0	24	***R. araiospora***	Also *R. subbotrytis*
120	0	2	***R. atractospora***	
121	0	6	***R. aurantiisiccescens***	OTU 122
122	0	1	***R. aurantiisiccescens***	OTU 121
123	0	1	***R. aurea***	OTU 124
124	0	2	***R. aurea***	OTU 123
125	0	1	***R. bataillei***	
126	0	1	***R. beninensis***	
127	0	1	***R. boreimaxima***	
128	0	1	***R. botrytis***	OTU 129,130
129	0	1	***R. botrytis***	OTU 128,130
130	0	1	***R. botrytis***	OTU 128,129
131	0	1	***R. brunneolilacina***	
132	0	10	***R. calvodistalis***	
133	0	12	***R. stricta***	Also *R. pseudogracilis*
134	0	4	***R. cartilaginea***	

135	0	3	***R. flavosaponaria***	Also *R. cauliflориformis*
136	0	1	***R. cedretorum***	
137	0	9	***R. celerivirescens***	Also *R. claviramulata*
138	0	3	***R. cistophila***	
139	0	1	***R. comitus***	
140	0	1	***R. concolor-stricta***	OTU 141
141	0	1	***R. concolor-stricta***	OTU 140
142	0	2	***R. conjunctipes***	OTU 143,144,145
143	0	3	***R. conjunctipes***	OTU 142,144,145
144	0	3	***R. conjunctipes***	OTU 142,143,145
145	0	1	***R. conjunctipes***	OTU 142,143,144
146	0	2	***R. coulterae***	OTU 147
147	0	1	***R. coulterae***	OTU 146
148	0	7	***R. cyaneigranosa***	
149	0	1	***R. flava***	OTU 150
150	0	1	***R. flava***	OTU 149
151	0	4	***R. flavescens***	
152	0	1	***R. flavicingula***	
153	0	1	***R. flavigelatinosa***	
154	0	2	***R. pallidissima***	Also *R. rubripermanens*
155	0	1	***R. flavobrunnescens***	
156	0	3	***R. ossolana***	Also *R. flavoides*
157	0	1	***R. flavoides***	
158	0	1	***R. foetida***	
159	0	3	***R. formosa***	OTU 160,161,162
160	0	3	***R. formosa***	OTU 159,161,162
161	0	1	***R. formosa***	OTU 159,160,162
162	0	2	***R. formosa***	OTU 159,160,161
163	0	2	***R. gelatiniaurantia***	
164	0	1	***R. graciloides***	
165	0	6	***R. gracilis***	OTU 96
166	0	1	***R. ichnusensis***	
167	0	1	***R. lignicolor***	
168	0	1	***R. largentii***	
169	0	1	***R. leptoformosa***	
170	0	4	***R. longispora***	
171	0	1	***R. lorithamnus***	
172	0	1	***R. luteoaurantica***	
173	0	1	***R. maculatipes***	
174	0	1	***R. magnifica***	
175	0	1	***R. mediterranea***	
176	0	1	***R. obtusissima***	
177	0	1	***R. ochrochlora***	
178	0	1	***R. pallidosaponaria***	
179	0	1	***R. parabotrytis***	
180	0	1	***R. paraconcolor***	
181	0	2	***R. pinicolor***	Also *R. stricta*
182	0	2	***R. rubella***	Also *R. polonica*
183	0	5	***R. praecox***	
184	0	1	***R. primulina***	
185	0	1	***R. pseudoflava***	
186	0	1	***R. pumila***	
187	0	2	***R. rasilospora***	
188	0	1	***R. rasilisporoides***	OTU 189
189	0	1	***R. rasilisporoides***	OTU 188
190	0	1	***R. rubiginosa***	
191	0	3	***R. rubribrunnescens***	OTU 192
192	0	1	***R. rubribrunnescens***	OTU 191
193	0	1	***R. rubricarnata***	
194	0	2	***R. rubrievanescens***	

195	0	3	***R. rubripermanens***	OT154
196	0	1	***R. rufescens***	
197	0	1	***R. sandaracina***	OTU 198,199
198	0	2	***R. sandaracina***	OTU 197,199
199	0	1	***R. sandaracina***	OTU 197,198
200	0	2	***R. sanguinea***	
201	0	1	***R. sinsinii***	
202	0	1	***R. karstenii***	
203	0	2	***R. spinulosa***	
204	0	1	***R. stricta***	OTU 133, 205
205	0	1	***R. stricta***	OTU 133, 204
206	0	3	***R. stuntzii***	
207	0	1	***R. subbotrytis***	OTU119
208	0	5	***R. suecica***	OTU 209
209	0	1	***R. suecica***	OTU 208
210	0	4	***R. testaceoflava***	
211	0	1	***R. thindii***	
212	0	1	***R. velocimutans***	
213	0	1	***R. verlotensis***	

A: OTUs defined on the basis of 97% nucleotide identity across the complete ITS region; B: Names in brackets indicate results of Blastn matches to partial ITS sequences in NCBI. Environmental sequences excluded; C: Instances where species were assigned to multiple OTUs or where multiple species names were assigned to the same OTUs are listed.
